# Adaptive HIV-Specific B Cell-Derived Humoral Immune Defenses of the Intestinal Mucosa in Children Exposed to HIV via Breast-Feeding

**DOI:** 10.1371/journal.pone.0063408

**Published:** 2013-05-21

**Authors:** Sandrine Moussa, Mohammad-Ali Jenabian, Jean Chrysostome Gody, Josiane Léal, Gérard Grésenguet, Alain Le Faou, Laurent Bélec

**Affiliations:** 1 Institut Pasteur de Bangui, Laboratoire des Rétrovirus-VIH, Bangui, Central African Republic; 2 Assistance Publique - Hôpitaux de Paris, Hôpital Européen Georges Pompidou, Laboratoire de Virologie, Paris, France; 3 Complexe Pédiatrique, Bangui, Central African Republic; 4 Unité de Recherches et d’Intervention sur les Maladies Sexuellement Transmissibles et le SIDA, Département de Santé Publique, Faculté des Sciences de la Santé de Bangui, Bangui, Central African Republic; 5 Faculté de Médecine Paris Descartes, Sorbonne Paris Cité, Paris, France; German Primate Center, Germany

## Abstract

**Background:**

We evaluated whether B cell-derived immune defenses of the gastro-intestinal tract are activated to produce HIV-specific antibodies in children continuously exposed to HIV via breast-feeding.

**Methods:**

Couples of HIV-1-infected mothers (n = 14) and their breastfed non HIV-infected (n = 8) and HIV-infected (n = 6) babies, and healthy HIV-negative mothers and breastfed babies (n = 10) as controls, were prospectively included at the Complexe Pédiatrique of Bangui, Central African Republic. Immunoglobulins (IgA, IgG and IgM) and anti-gp160 antibodies from mother’s milk and stools of breastfed children were quantified by ELISA. Immunoaffinity purified anti-gp160 antibodies were characterized functionally regarding their capacity to reduce attachment and/or infection of R5- and X4- tropic HIV-1 strains on human colorectal epithelial HT29 cells line or monocyte-derived-macrophages (MDM).

**Results:**

The levels of total IgA and IgG were increased in milk of HIV-infected mothers and stools of HIV-exposed children, indicating the activation of B cell-derived mucosal immunity. Breast milk samples as well as stool samples from HIV-negative and HIV-infected babies exposed to HIV by breast-feeding, contained high levels of HIV-specific antibodies, mainly IgG antibodies, less frequently IgA antibodies, and rarely IgM antibodies. Relative ratios of excretion by reference to lactoferrin calculated for HIV-specific IgA, IgG and IgM in stools of HIV-exposed children were largely superior to 1, indicating active production of HIV-specific antibodies by the intestinal mucosa. Antibodies to gp160 purified from pooled stools of HIV-exposed breastfed children inhibited the attachment of HIV-1NDK on HT29 cells by 63% and on MDM by 77%, and the attachment of HIV-1JRCSF on MDM by 40%; and the infection of MDM by HIV-1JRCSF by 93%.

**Conclusions:**

The intestinal mucosa of children exposed to HIV by breast-feeding produces HIV-specific antibodies harbouring in vitro major functional properties against HIV. These observations lay the conceptual basis for the design of a prophylactic vaccine against HIV in exposed children.

## Introduction

The UNAIDS estimated that more than 330,000 (280,000–380,000) children were newly infected by human immunodeficiency virus type 1 (HIV-1) through mother-to-child transmission (MTCT) worldwide in 2011, with the majority (>90%) occurring in sub-Saharan Africa [1]. The majority of MTCT occurs during pregnancy and birth. In addition, postnatal transmission of HIV-1 from HIV-infected mother to her child through prolonged breast-feeding is well recognized, and may account for one-third to half of new infant HIV-1 infections worldwide [2]–[10]. While studies of maternal or infant antiretroviral therapy during the period of breast-feeding have shown substantial potential for reduction of infant HIV infections [11]–[14], postnatal virus transmissions may continue to occur even in the setting of optimal antiretroviral prophylaxis [15].Therefore, development of immunologic strategies to reduce HIV transmission via breast milk remains important for improving survival of babies born to HIV-infected mothers in the developing world.

Despite the babies daily exposure via their oral and gastrointestinal mucosae to high amounts of cell-associated and cell-free HIV-1, estimated to be more than 700,000 viral particles per day [16], HIV acquisition in exposed breastfed children occurs infrequently. The overall probability of transmission via breast-feeding was estimated to range from to 0.050 [17] to 0.064 [18] percent per liter of breast milk ingested. Consumption of 0.5–1.0 liter of breast milk daily provides continuous exposure to potentially infectious virus through the oral cavity and the gastrointestinal mucosa. On the other hand, less than 10% of babies born to HIV-infected women and breastfed during the first 6 months of life become infected postnatal [19], indicating low efficiency of breast milk transmission which is in contrast with the daily exposure to high amount of infectious viral particles. The low frequency of breast-feeding acquisition suggests that anti-infective factors in breast-feeding HIV-infected mothers as well as in HIV exposed breastfed children are involved [20]. The fact that the majority of breastfed babies of HIV-infected mothers remain uninfected even after several months of breast-feeding constitutes one of the major paradoxes of HIV transmission via breast milk [21].

The majority of exposures to HIV-1 in breastfed children is across oral mucosa, tonsillar tissue and gastrointestinal mucosa, which are immunocompetent tissues, belonging to the afferent branch of the mucosa-associated lymphoid tissue (MALT) [22]. Induction of mucosal immunity against HIV following prolonged child exposure to infected breast milk is an attractive hypothesis [21], [23], [24]. Previous studies showed that HIV-1-uninfected babies exposed to HIV via breast-feeding may develop HIV-1-specific salivary IgA [25] as well as systemic HIV-specific CD8 cytotoxic immune responses [26].Overall, these observations suggest that the specific humoral and cellular immune defenses are activated in breastfed children. The confirmation that the child exposed to HIV through breast-feeding actually develops protective specific immunity against the acquisition of the virus could have major importance for the demonstration of immunological correlates of protection, and for the design of a prophylactic vaccine.

The aim of the present study was to evaluate whether B cell-derived immune defenses of gastro-intestinal tract are activated to produce HIV-specific antibodies in breastfed children continuously exposed to HIV via breast-feeding. For that purpose, HIV-specific antibodies were first detected in immunoglobulins purified from stools of breastfed children and characterized immunochemically. Furthermore, their functional properties were assessed by their capability to hamper in vitro the attachment of the virus to intestinal epithelial cells and monocyte-derived macrophages (MDM), and further by their aptitude to modulate negatively HIV production in cell culture.

## Materials and Methods

### Inclusion of Mothers and their Breastfed Babies

Couples of HIV-1-infected mothers and their breastfed babies were consecutively recruited at the Complexe Pédiatrique, the principal health care clinic for HIV-infected children held in Bangui, the capital city of the Central African Republic [27]–[29].The study was formally approved by the Scientific Committee of the Faculté des Sciences de la Santé (“FACSS”) of Bangui (so-called “Comité Scientifique Chargé de la Validation des Protocoles d’Etudes et des Résultats”/”CSCVPER”) (agreement 2UB/FACSS/CSCVPER/05), constituting the National Ethical Committee. Informed written consent was obtained from mothers for themselves and on behalf of their respective child participating in the study. All HIV-infected mother and their babies received care, and when indicated antiretroviral treatment, according to the WHO recommendations for the management of HIV infection in resource-limited settings [30], [31]. All HIV-infected children received co-trimoxazole as prophylaxis against opportunistic infections [32].

Inclusion criteria for HIV-infected mother-child couples in the study were as follows: i) HIV-infected mother; ii) baby born from HIV-infected mother and exposed to HIV by exclusive breast-feeding from birth; iii) early diagnosis of HIV infection or non HIV infection at time of sampling in babies born from HIV-infected mother by molecular virological diagnosis; iv) mother and babies care according to the national guidelines. The exclusion criteria were: i) HIV diagnosis not formally established; ii) lack of informal consent; iii) recent (<1 month) past history of gastro-enteritis in breastfed children. Note that at time of period inclusion, interruption of antiretroviral drugs availability throughout the country unfortunately has not allowed to treat any HIV-infected mothers or children for a period of at least one month before inclusion.

Ten healthy volunteer HIV-seronegative breast-feeding women and their breastfed HIV-non infected babies from the same setting were also included as negative controls.

### Collection and Processing of Clinical Samples

K3-EDTA-blood samples were obtained from study mothers and their babies by venipuncture in Vacutainer tubes (Becton Dickinson, Franklin Lakes, NJ, USA). The plasma was separated after centrifugation at 1000×g for 10 minutes, and aliquots were kept frozen at −80°C within 2 hours after sampling until processing. Of note, maternal milk and infant stool samples were collected at the same time during the same visit.

Milk samples (10 ml) were collected manually, and then centrifuged at 9,300×*g* for 20 minutes at +4°C, allowing separation of the cellular, supernatant and lipid fractions, as previously described [33]. The pellet and fat layer were discarded, and the supernatant was collected, and aliquots were stored at −80°C until processing.

Stool samples from babies were collected at room temperature, and then mixed with “cold buffer” conserved at +4°C. This buffer is constituted by phosphate buffered saline (PBS, pH = 7.3) containing 1 mM of the serine proteases inhibitor phenyl methyl sulphonyl fluoride (Sigma Aldrich, St-Louis, MO, USA) (10% wt/vol). The mixture was vortexed for at least 1 minute, and then let for 10 minutes at room temperature, and then centrifuged at 2,700×*g* for 15 minutes at +4°C. Aliquots of resulting supernatants were stored at −80°C until use.

### Diagnosis of HIV Infection

Molecular diagnosis of HIV in children born from HIV-infected mother was carried out by assessing the circulating plasma HIV-1 RNA load, as previously shown [34]. HIV-1 RNA load in plasma from babies was measured by the Generic HIV-1 RNA quantification assay (Biocentric, Bandol, France) using the ABI PRISM 7000 real-time PCR system (Applied Biosystems, California, USA), as previously described [35].

### Antibodies and Reagents

Anti-human Fc fragment of IgA (α-chain specific), anti-human Fc fragment of IgG (γ-chain specific), and peroxidase (PO)-labeled anti-human IgG(γ-chain specific) were obtained from Pierce (Rockford, IL, USA). Anti-human Fc fragment of IgM (µ-chain specific), biotinylated anti-human IgM (μ-chain specific), and anti-human lactoferrin (Lf), were obtained from Sigma Aldrich. Horseradish peroxidase (HRPO)-labeled streptavidin was obtained from Immunotech (Marseille, France). The anti-human IgA-PO-conjugated, the anti-human Lf-PO-conjugated and anti-human F(ab’)2-PO-conjugated were from our laboratory. Anti-HIV-1 gp120 monoclonal antibodies IgG2G12 and IgG1B12 were obtained from the AIDS Reagent Program, Division of AIDS, NIAID, NIH [36].

The gp160 antigen consisted of a purified preparation of baculovirus-expressed recombinant gp160 (rgp160) derived from the envelope of the HIV-1MN/LAI strain (kindly provided by Aventis-Pasteur, Paris, France). Recombinant human macrophage colony-stimulating factor (rhM-CSF) was from R&D Systems Europe (Abingdon, United Kingdom). RPMI 1640 (with L-glutamine) was provided by Cambrex (Verviers, Belgium), and penicillin and streptomycin were provided by Invitrogen (Paisley, United Kingdom). Medium for separation of lymphocytes (MSL) was obtained from PAA (Les Mureaux, France), and fetal calf serum (FCS) was provided by Eurobio (Les Ulis, France). Sepharose® 4B was obtained from Sigma Aldrich and anti-human F(ab’)2 from Jackson Immunoresearch (West Grove, PA, USA). The HIV-1 p24 antigen capture enzyme-linked immunosorbent assay (ELISA) was obtained from Innogenetics(Gent, Belgium).

Polyclonal anti-gp160 IgG was purified from a pool of sera from HIV-1-infected patients (laboratoire de virologie, Hôpital Européen Georges Pompidou, Paris, France), to be used as positive control in immunochemical assays detecting HIV-specific antibodies, as previously described [37], [38].

A stock solution of IVIg (50 mg/ml; 0.3 mM) corresponding to pooled normal IgG obtained from plasma of healthy donors was prepared in PBS and dialysed twice against large volume of PBS at 4°C to remove the stabilizing agents, as previously described [39]. IVIg contained mostly monomeric IgG (>95%) and was used as negative control in functional inhibitory assays.

### Cells

Peripheral blood mononuclear cells (PBMC) were isolated from buffy coats of healthy adult donors by Ficoll density gradient centrifugation on MSL, as previously described [40]. Blood samples from HIV-negative healthy donors for functional assays were collected at the French Blood Establishment, Paris, France. Informed written consents from all subjects were obtained before blood sampling. The percentage of monocytes was determined by flow cytometry (FACS Calibur, Becton Dickinson, NJ, USA) using forward scatter and side scatter properties (FSC/SSC). PBMC were re-suspended in RPMI 1640 medium supplemented with glutamine, penicillin (100 IU/ml) and streptomycin (100 µg/ml). Cells were seeded into 24 well-plates (Costar, Cambridge, MA) at the concentration 1×10^6^ adherent cells/ml and incubated at 37°C for 45 minutes. Non adherent cells were removed by 4 washes. Adherent monocytes were incubated in RPMI medium with 10% FCS, glutamine, and antibiotics in the presence of 10 ng/ml rhM-CSF (10 ng/ml) to differentiate to macrophages, as previously described [41]. The relative concentration of rhM-CSF improve cell viability and maintained a neutral environment with respect to activation marker quantitative expression (HLA-DR, CD14, CD16), which remained similar to that of MDM cultured in medium alone [41]. Half of the medium, including all supplements, was replaced every 3 days. After 7 days of culture, adherent cells corresponding to the macrophages-enriched fraction were harvested, washed, and used for subsequent experiments [41], [42]. At the time of collection, MDM were more than 90% pure, expressing by flow cytometry analysis (CellQuest software, Becton Dickinson) CD4^+^, CXCR4^low^, CCR5^high+^, CD14 (73%) and CD11b (70%) (data not shown).

The HT-29 human colorectal epithelial cells line was provided by the AmericanType Culture Collection (ATCC HTB-38, Manassas, VA, USA). Cells were grown in RPMI 1640 medium complemented with 10% FCS, penicillin (100 IU/ml) and streptomycin (100 µg/ml). The HT-29 cells were CD4^−^, DC-SIGN^−^, CXCR4^high+^, CCR5^low+^ and GalCer^high+^ (data not shown).

### HIV Strains

Primary X4-tropicstrain HIV-1_NDK_ and R5-tropic strain HIV-1_JR-CSF_ were a gift of Prof. F. Barré-Sinoussi (Institut Pasteur,Paris, France). Stocks of the HIV-1NDK strains were produced on IL-2-activated peripheral blood lymphocytes (PBL) of healthy donors. The HIV-1_JR-CSF_ strain was amplified in MDM cultures. The virus produced was clarified by centrifugation, and the HIV p24 concentration was determined by capture ELISA, and stored at −80°C. Tissue culture infective dose 50% (TCID50) of each stock was calculated according to the Kärber formula [43], 1 ng of p24 antigen corresponding to 1000 TCID50, as previously shown [38].Primary strains are thought to be representative of viral strains not adapted to their microcellular environment. Furthermore, monocytotropic (R5+) [44], [45] and to a lesser extent lymphocytotropic (X4+) [45] HIV-1 strains are present in breast milk, and may participate to HIV mucosal crossing in exposed receptive baby.

### Quantification of Total IgA, IgG and IgM Antibodies in Breast Milk and Stool Samples

Total immunoglobulins (IgA, IgG and IgM) in the mother’s milk and in non-purified children’s stools were quantified by asymmetrical ELISA, as previously described [46]. Briefly, plastic plates were coated with goat anti-human α chain, γ chain or µ chain (all at 3 µg/ml) in PBS overnight at +4°, prior to washing with PBS/0.1% Tween, and saturated with PBS/1% skimmed milk. Serial dilutions of breast milk and stools supernatants were then added for 1 hour at +37°C. After further washes, goat anti-human F(ab’)2 (2 µg/ml) coupled with peroxidase was added for 1 hour at 37°C. After extensive washes, the peroxidase activity was revealed with *o*-phenylenediamine (OPD) (Sigma Aldrich), and the optical density (OD) was read at 492 nm. Quantification of each immunoglobulin class was assessed by extrapolation from standard curves obtained by serial dilutions of a pool of 20 samples of normal human colostrum, as described elsewhere [37], [46].

### Detection of Anti-gp160 Antibodies in Breast Milk and Stool Samples and Calculation of their Specific activities

The detection of anti-gp160 antibodies in breast milk and children’s stools was assessed by indirect ELISA, in part as previously described [33]. Briefly, plastic plates were coated overnight at +4° with rgp160 (1 µg/ml) in PBS. The plates were washed with PBS/0.1% Tween prior to saturation with PBS/1% skimmed powder milk. Dilutions of breast milk and stools supernatants were then added, and incubated for 1 hour at +37°C. After washing, peroxidase-labelled goat antibodies (2 µg/ml) to human F(ab’)2, IgA or IgG, were added for 1 hour at +37°C prior to addition of peroxidase substrate (OPD); for IgM antibodies, biotinylated goat antibodies to IgM (0.5 µg/ml) were first added for 1 hour at +37°C, followed by streptavidin-HRPO for 10 minutes at +37°C, prior to addition of peroxidase substrate (OPD). The cut-offs of F(ab’)2, IgA or IgG positivity for milk or stools samples were defined as the mean OD plus 2 standard deviations (SD) of the values obtained with breast milk or children’s stools samples from the HIV-negative controls. The cut-off of IgM positivity for milk or stools samples was defined as the mean OD plus 3 SD of the values obtained with breast milk or children’s stools samples from the HIV-negative controls. Finally, the levels of IgA, IgG and IgM to gp160 in milk and stools samples positive for anti-gp160 antibodies were expressed in arbitrary OD (at 492 nm) units (AU).

The specific activities (SA) of antibodies to gp160 of the IgA isotype (SAIgA to gp160) in breast milk (M) and stools (S) were expressed as the ratio of AU (OD reactivity) per µg of total IgA, according to the following formulae:
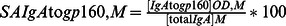


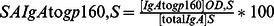



Similarly were calculated the SA of IgG to gp160 in milk (SAIgG to gp160,M) and stools (SAIgG to gp160,S), as well as the SA of IgM to gp160 in milk (SAIgM to gp160,M) and stools (SAIgM to gp160,S).

### Levels of Lactoferrin in Breast Milk and Stool Samples

Lf in mother’s milk and in children’s stools was measured by symmetrical ELISA. In brief, plastic plates were coated with anti-human Lf (1 µg/ml) in PBS overnight at +4°C. The plates were washed with PBS/0.1% Tween and saturated with PBS/1% gelatin. Serial dilutions of mother’s milk, children’s stools supernatants and human Lf in PBS (standard) were then added in the plates and incubated for 1 hour at +37°C. After further washes, goat anti-human Lf antibody coupled with peroxidase was added for 1 hour at +37°C before addition of substrate (OPD) and quantification of peroxidase activity by OD at 492 nm. Quantification of milk or stool Lf was assessed by extrapolation from the standard curve obtained by serial dilutions of known amount of human Lf.

### Evaluation of Intestinal Production of Stool Immunoglobulins and Anti-gp160 Antibodies

Lf is an iron-binding glycoprotein secreted from many epithelial cells into most exocrine fluids, particularly in breast milk [47]. Lf is thought to be poorly or not secreted by the intestinal mucosa of the new born, and fecal Lf is mainly originating from breast milk in breastfed infant [48].Indeed, the mean levels of fecal Lf reported in the literature in bottle fed infants around 0.5 mg per day [49], thus nearly 90% less than those usually reported in breast milk [50], and around 12.5 mg per day in breastfed children, thus 25-fold more than in bottle fed infants [49]. The fecal levels of Lf increase in case of gastro-intestinal inflammatory or infectious diseases, such as inflammatory bowel diseases, Crohn's disease, ulcerative colitis and gastro-enteritis [47], [51]. In breastfed children, fecal Lf is likely originating from breast milk intake, corresponding to undegraded Lf passively seeped into the intestinal chyle, and to a lesser extent from intestinal production, normally negligible in absence of intestinal inflammation [52]. Similarly, it is possible to consider that fecal immunoglobulins in breastfed children correspond to undegraded immunoglobulins coming from breast milk intake as well as to intestinal immunoglobulin production.

These latter considerations prompt us evaluating intestinal production of stool immunoglobulins in breastfed children, taken into account a simplified relationship between breast milk immunoglobulins ([Ig]M) and Lf ([Lf]M), and stool immunoglobulins ([Ig]S) and Lf ([Lf]S).

Thus, stool immunoglobulins is the sum of undegraded immunoglobulins from breast milk ([Ig]S,bm), and intestinal immunoglobulins production ([Ig]S,i):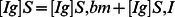.[Ig]S,bm corresponds to a fraction α of [Ig]M :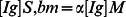.Finally, 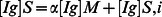.

Similarly, stool Lf is the sum of undegraded Lf from breast milk ([Lf]S,bm), and intestinal Lf production ([Lf]S,i):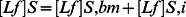. [Lf]S,bm corresponds to a fraction α' of [Lf]M :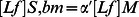. If one hypothesizes that 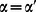, because immunoglobulins and Lf are glycoproteins similarly degraded in the intestinal chyle, and that the intestinal production of Lf is negligible in the absence of gastro-intestinal inflammation or gastro-enteritis 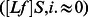,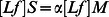, and 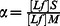.

Thus,
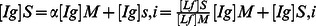
and



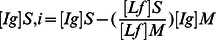
(1)In case of intestinal/fecal production of immunoglobulins,
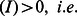



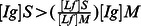
, and



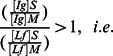





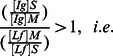





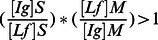



In opposite, in case of intestinal/fecal immunoglobulins exclusively provided from breast milk, 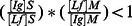.

Taken together, the following relative ratio of excretion (RRE).



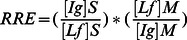
may be used to evaluate the relative fecal/intestinal production of immunoglobulins in feces from breastfed children, by reference to Lf as breast milk intake factor, with the hypotheses that immunoglobulins and Lf are glycoproteins similarly degraded within intestinal chyle, and that the intestinal production of Lf is negligible in the absence of gastro-intestinal inflammation or gastro-enteritis in breastfed children. When this formula is applied to HIV-specific antibodies, a RRE is superior to 1 in baby exposed to HIV means that HIV-specific antibodies evidenced in stools are not only originating from a passive ingestion of breast milk antibodies, but rather indicates the infant intestinal mucosa likely secretes actively HIV-specific antibodies.

Finally, in order to assess whether HIV-exposed children secrete total and/or HIV-specific antibodies during breast-feeding in their intestinal mucosa, we calculated the RRE of stool IgA (RREIgA,S), IgG (RREIgG,S), and IgM (RREIgM,S) by reference to Lf, according to the following formulae:



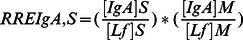





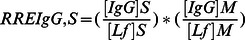





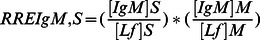



Similarly, the RRE of stool gp160-specific IgA (RREIgA to gp160,S), IgG (RREIgG to gp160,S), and IgM (RREIgM to gp160,S), were calculated by reference to Lf, according to the following formulae:



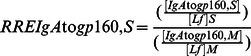





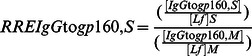





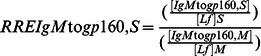



Thus,
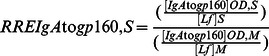


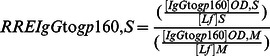


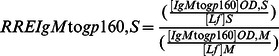



The RRE of stool specific antibodies to gp160 of a given isotype could be calculated only when the denominator is not zero, thus when HIV-specific antibodies of the same isotype is detected in corresponding breast milk.

### Immunoaffinity Purification of Total Antibodies from Pooled Stools of Breastfed Children

Total immunoglobulins were purified by immunoaffinity, as previously described [46], [53]. In brief, the anti-human F(ab’)2 was first coupled to Sepharose® 4B. Pool of stool samples (supernatants) from HIV exposed non HIV-infected (group I) and HIV-infected (group II) children, as well as from HIV non exposed control babies were afterwards incubated with the matrix overnight at +4°C before extensive washing of the column with PBS until the OD of the effluent reached a value of 0.001 at 280 nm. The column was then eluted with 0.2 M glycine-HCl, pH 2.5. The eluate was rapidly neutralized with 1 MTris-HCl, pH 8.3, and dialysed against PBS overnight.

The isotype (IgA, IgG and IgM) composition of affinity-purified anti-human F(ab’)2 was measured by ELISA. Plates were coated with goat anti-human α-chain, γ-chain or µ-chain (all at 3 µg/ml) in PBS overnight at +4°C, prior to washing with PBS/0.1% Tween and then saturated with PBS/1% skimmed milk. Serial dilutions of pooled stools immunopurified anti-F(ab’)2 antibodies were then added for one hour at +37°C. After further washes, goat anti-human F(ab’)2 (2 µg/ml) coupled with peroxidase was added for 1 hour at +37°C. After extensive washes, substrate (OPD) was added and peroxidase activity was determined by OD at 492 nm. A pool of normal human sera with known levels of IgA, IgG and IgM was used to obtain standard curves.

### Detection HIV-specific F(ab’)2 in Total Immunoaffinity Purified Stool Immunoglobulins

Plastic plates were coated overnight at +4°C with rgp160 (1 µg/ml) in PBS. The plates were washed with PBS/0.1% Tween prior to saturation with PBS/1% skimmed milk. Serial dilutions of antibodies purified from pooled stools were then added and incubated for 1 hour at +37°C. After washing, peroxidase-labelled goat anti-human F(ab’)2 (2 µg/ml) were added for 1 hour at +37°C prior to addition of peroxidase substrate (OPD). The levels of F(ab’)2 to gp160 in stools were expressed in arbitrary OD (at 492 nm) units. The cut-off of positivity was defined as the mean OD plus 2 SD of the values obtained with breast milk samples from the HIV-negative controls. The SA of purified F(ab’)2 to gp160 in pooled stools from groups I and II were calculated as above as the ratio of AU (OD reactivity at 492 nm) per µg of total 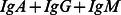 in each pool.

### Inhibition of HIV-1 Attachment to HT29 Cells and Monocyte-derived Macrophages by Purified Stools Immunoglobulins

HT29 cells or MDM (250,000 cells/well) were pre-incubated with pooled stools immunoglobulins (30 or 50 µg/ml) purified from HIV exposed group I and group II children for 1 hour at +37°C before addition of 10 ng/ml HIV-1JRCSF p24 antigen and HIV-1NDKp24 antigen for 1 hour at +37°C. Cells were then extensively washed and lysed with 0.5% Triton, and HIV-1 p24 antigen levels were measured by ELISA. As attachment inhibition positive control, 50 µg of Lf was added to cells prior to the incubation with HIV, as previously described [54]. As attachment inhibition negative controls, IVIg and total immunoglobulins purified by immunoaffinity from pooled stools of HIV non exposed control babies (50 µg/ml) were added to HT29 cells prior to the incubation with HIV. Each experiment was carried out in triplicate.

### HIV-1 Infection Inhibition of Monocyte-derived Macrophages by Purified Stools Immunoglobulins

MDM were washed 2 times after 6 days of differentiation, and seeded into 96-well culture plates (250,000 cells/well). At day 7, pooled stools antibodies purified from HIV exposed group I and group II children at 50 µg/ml were added to cells for 1 hour at +37°C before addition of HIV-1JRCSF and HIV-1NDK (10 ng/ml of p24 antigen). The cells were further incubated for 3 hours at +37°C in a 5% CO2 atmosphere. After 4 washes to remove exceeding virus, cells were cultured for 3 and 6 days. As inhibitory positive control, monoclonal antibody IgG2G12 (20 µg/ml) was added to MDM for 1 hour at +37°C before addition of HIV. As negative controls, IVIg and total immunoglobulins purified from pooled stools of HIV non exposed control babies (50 µg/ml) were added to MDM prior to the incubation with HIV. The levels of virus replication were estimated by HIV-1 p24 antigen ELISA on the supernatant of cells culture. Each experiment was carried out in triplicate.

### Statistical Analysis

Levels of immunoglobulins, Lf, HIV-specific antibody SA, and RRE were expressed as mean±standard error. Functional tests were expressed as percentage ± standard error. The non-parametric Mann-Whitney *U* test was used for statistical analyses, using GraphPad Prism 5.0 (San Diego, California, US) statistical software. A P-value <0.05 was considered as significant.

## Results

### Molecular Diagnosis of HIV Infection in Breastfed Infants

The direct detection of circulating HIV RNA allows early diagnosing of HIV infection in children aged less than 12 months born from HIV-infected mother [30], [55], [56]. Among 36 couples of HIV-1-infected mothers and their breastfed babies consecutively recruited, 25 (69%) babies were negative for plasma HIV-1 RNA, and thus HIV non infected, whereas 11 (31%) were positive, and thus HIV-infected. We further selected mother-child couples whose biological samples were in sufficient quantity for study experiments, including 8 couples of HIV-1-infected mothers and their breast milk exposed non infected babies (group I), and 6 couples of HIV-1-infected mothers and their breastfed infected babies (group II). The main characteristics of the 14 study mother-child couples are depicted in the Table 1. The mean duration of exclusive breast-feeding in study couples was 4.5 months, without difference in group I (mean duration: 4.6 months) and group II (mean duration: 3.5 months). All but one (children #I) was asymptomatic for HIV infection. At time of sampling, none of the mothers had symptoms of mammary inflammation, and none of the children showed gastro-intestinal symptoms or infectious diarrhoea.

**Table 1:** Main characteristics of study mother-child couples, including 8 couples of HIV-1-infected mothers and their breastfed non HIV-infected babies (group I), and 6 couples of HIV-1-infected mothers and their breastfed HIV-1-infected babies (group II).

			Mothers			Breastfed infants			
Study Groups	Couple	Exclusive breast-feeding duration(month)	Age(year)	WHO clinicalstage£	Mastitis	HIV-1 RNA load(log/ml)*	WHO clinical stage$	Co-trimoxazoleprophylaxis	Gastro-intestinal symptoms
**Group I**(n = 8)	#A	3.5	34	1	No	Undetectable	NA	NA	No
	#B	4	25	1	No	Undetectable	NA	NA	No
	#C	6	34	2	No	Undetectable	NA	NA	No
	#D	6	23	1	No	Undetectable	NA	NA	No
	#E	7	20	1	No	Undetectable	NA	NA	No
	#F	3	24	1	No	Undetectable	NA	NA	No
	#G	2	35	1	No	Undetectable	NA	NA	No
	#H	2.5	25	1	No	Undetectable	NA	NA	No
**Group II**(n = 6)	#I	4	32	1	No	6.7	2	Yes	No
	#II	3	27	1	No	5.1	1	Yes	No
	#III	4	26	1	No	1.8	1	Yes	No
	#IV	2	24	1	No	2.2	1	Yes	No
	#V	4	25	1	No	4.6	1	Yes	No
	#VI	1.5	30	2	No	6.3	1	Yes	No

£ World Health Organization (WHO) clinical staging of HIV/AIDS for adults and adolescent with established HIV infection according to the 2010-revised WHO recommendations for HIV care in in babies and children for resource-limited settings [31];

$ WHO clinical staging of HIV/AIDS for children with established HIV infection according to the 2010-revised WHO recommendations for HIV care in babies and children for resource-limited settings [30]; CD4 T cell count in infants were not available because the national AIDS programme has focused on universal treatment for infants less than 12 months, independently of their clinical or immunological status;

* Plasma HIV-1 RNA load was measuredby the Generic HIV-1 RNA quantification assay (Biocentric, Bandol, France), which threshold of detectability is 300 copies/ml [35].The molecular diagnosis of HIV infection was carried out at time of sampling; the timing of HIV infection in study children could not be assessed.

NA: Not applicable.

### Quantification of Total IgA, IgG and IgM in Breast Milk and Children’s Stools

**Figure 1:**
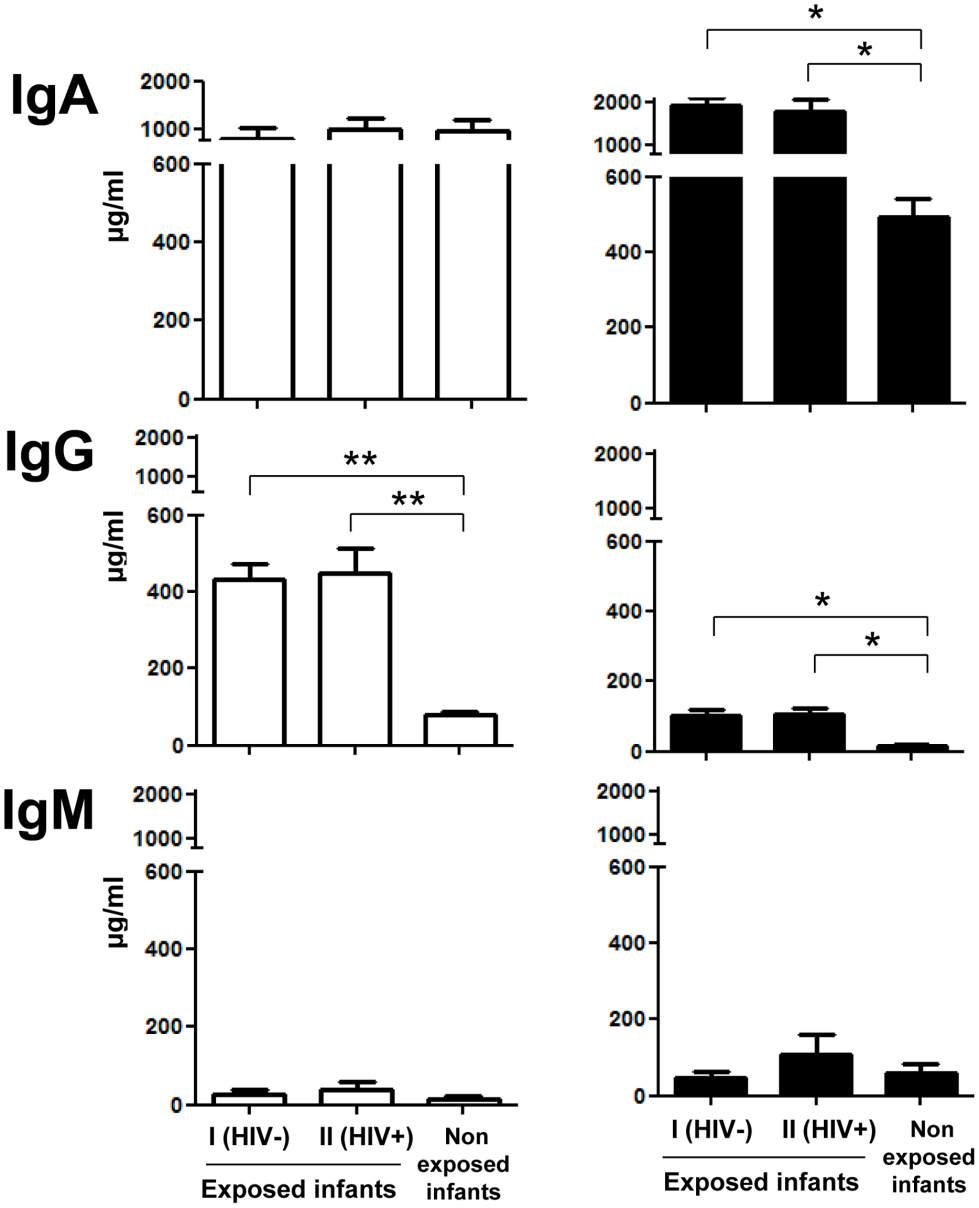
Levels of total IgA, IgG and IgM in breast milk (white bars in left) and children’s stools (black bars in right) samples from 8 HIV-1-infected mothers and their breast milk exposed non HIV-infected babies (group I), 6 couples of HIV-1-infected mothers and their breastfed HIV-infected babies (group II), and 10 healthy HIV-negative breast-feeding women and their breastfed non HIV-infected babies as negative controls. The mean concentrations of milk IgA were higher than those of IgG or IgM in HIV-infected mothers as in HIV-negative mothers. The levels of milk IgG was 5.5 higher in HIV-infected mothers than in HIV-negative mothers. The levels of stool IgA and IgG in babies born from HIV-infected mothers were higher than those of HIV-negative control babies. Immunoglobulins levels are expressed in µg/ml ± standard error. The stars indicate the significant differences by comparison to HIV-negative control samples (* P<0.01).

The results of IgA, IgG and IgM levels in mothers’ milk and children’s stools from couples of groups I and II, and from HIV-negative control couples, are depicted in the Figure 1.

The mean concentrations of milk IgA in HIV-infected mothers were higher than those of IgG, which were also higher than those of IgM (milk IgA: 808±241 µg/ml in group I and 1025±212 µg/ml in group II; milk IgG: 433±41 µg/ml in group I and 447±66 µg/ml in group II; milk IgM: 28±11 µg/ml in group I and 40±20 µg/ml in group II; P<0.01 for all comparisons). In HIV-negative control couples, the mean concentration of IgA in milk (969±240 µg/ml) was higher than those of milk IgG (78±10 µg/ml) and IgM (15±8 µg/ml), which were of similar levels. Interestingly, the levels of milk IgG was 5.5 higher in HIV-infected mothers than in HIV-negative control mothers (P<0.01).

The mean concentrations of stool IgA in babies born from HIV-infected mothers were higher than those of IgG, which were higher than those of IgM (stool IgA: 1909±179 µg/ml in group I and 1767±287 µg/ml in group II; stool IgG: 104±15 µg/ml in group I and 108±16 µg/ml in group II; stool IgM: 48±16 µg/ml in group I and 108±58 µg/ml in group II; P<0.01 for all comparisons excepting the comparison between stool IgG and IgM in group II). In HIV-negative control couples, the mean concentration of stool IgA (494±48 µg/ml) was higher than those of IgM (58±27 µg/ml) (P<0.01), which were moderately higher than those of IgG in stool (17±3 µg/ml) (P<0.05). The levels of stool IgA and IgG in babies born from HIV-infected mothers (groups I and II) were higher than those of HIV-negative control babies (P<0.01). The levels of stool IgM were higher than those of HIV-negative babies only in group II (P<0.01).

### HIV-specific F(ab’)2, IgA, IgG and IgM in Breast Milk and Children’s Stools

The detection of antibodies directed to gp160 in breast milk and children’s stool samples and their corresponding calculated SA are presented in the Table 2.

**Table 2:** Detection of F(ab’)2, IgA, IgG and IgM to gp160 and specific activities (SA) of IgA, IgG and IgM to gp160, in milk and children’s stools from 8 couples of HIV-1-infected mothers and their breast milk exposed non HIV-infected babies (group I), and 6 couples of HIV-1-infected mothers and their breastfed HIV-1-infected babies (group II).

		Breast milk		Children’s stools	
		Group I(n = 8)	Group II(n = 6)	Group I(n = 8)	Group II(n = 6)
**F(ab’)2 to gp160** α	Detection (n; %)*	8 (100%)	6 (100%)	8 (100%)	6 (100%)
**IgA to gp160** α	Detection (n; %)*	8 (100%)	6 (100%)	7 (87%)	6 (100%)
	SAIgA to gp160**	2.1±0.2	1.6±0.2	0.9±0.2	1.7±0.6
**IgG to gp160** α	Detection (n; %)*	8 (100%)	6 (100%)	8 (100%)	6 (100%)
	SAIgG to gp160**	2.7±0.1	2.6±0.4	12.0±1.5	12.2±1.8
**IgM to gp160** β	Detection (n; %)*	0 (0%)	2 (33%)	3 (37%)	2 (33%)
	SAIgM to gp160**	NA	0.8±0.5	0.5±0.3	0.3±0.2

* n = number of positive samples, *e.g.* whose optical density (OD) by ELISA was above the calculated cut-offs of positivity for breast milk or stool samples;

** The specific activities of antibodies to gp160 were expressed as arbitrary units of OD reactivity at 492 nm per µg of total immunoglobulin of a given isotype (mean±standard error);

α F(ab’)2, IgA and IgG to gp160 were detected by indirect ELISA using recombinant gp160 as antigen, and peroxidase-labelled goat antibodies to human F(ab’)2, total IgA or IgG, as conjugates. The cut-offs of F(ab’)2, IgA or IgG positivity for milk or stool samples were defined as the mean OD at 492 nm plus 2 standard deviations of the values obtained with breast milk or children’s stool samples from the HIV-negative controls;

β IgM to gp160 were detected by biotine-streptavidine amplified indirect ELISA using recombinant gp160 as antigen, and biotinylated goat antibodies to IgM as conjugate, followed by horseradish peroxidase-labeled streptavidin revelation. To avoid the risk of false positivity, the cut-off of IgM positivity for milk or stool samples was defined as the mean OD plus 3 standard deviations of the values obtained with breast milk or children’s stool samples from the HIV-negative controls.

NA: Not applicable.

F(ab’)2, IgA and IgG to gp160 were present in all breast milk samples of non-transmitting (group I) and transmitting (group II) mothers, with specific anti-gp160 activity of 1.8±0.2 AU/µg for IgA and 2.6±0.4 AU/µg for IgG. The mean SA of IgG antibodies to gp160 was slightly higher than that of IgA to gp160 in milk samples from group I (P<0.05) as from group II (P<0.02). HIV-specific IgM were detected in only 2 to 14 (14%) breast milk samples, including one-third of breast milk samples from women of group II. Thus, IgA and IgG represented the major isotypes of HIV-specific antibodies in breast milk samples from HIV-1-infected mothers.

F(ab’)2, IgA, IgG or IgM to gp160 were present in nearly all stool samples of children’s of the non-transmitting (group I) and transmitting (group II) mothers, with specific anti-gp160 activity of 1.3±0.3 AU/µg for IgA, 12.1±1.6 AU/µg for IgG, and 0.4±0.3 AU/µg for IgM. The mean SA of IgG antibodies to gp160 was 12 and 7 times higher than that of IgA to gp160 in stool samples from group I and group II, respectively (P<0.01); and 24 and 40 times higher than that of IgM to gp160 in stool samples from group I and group II, respectively (P<0.001). The mean SA of IgA antibodies to gp160 was 2 and 5 times higher than that of IgM to gp160 in stool samples from group I and group II, respectively (P<0.01). Thus, HIV-specific antibodies of the IgA and IgG isotypes could be generally detected in stool samples from breastfed children whose mothers are HIV-1-infected, the IgG isotype being the most important.

No antibodies to gp160 could be detected in the milk’s mothers and stools ‘children samples from HIV-negative control mothers and babies.

### Relative Ratios of Excretion by Reference to Lactoferrin of Stool Immunoglobulins and Anti-gp160 Antibodies

**Figure 2:**
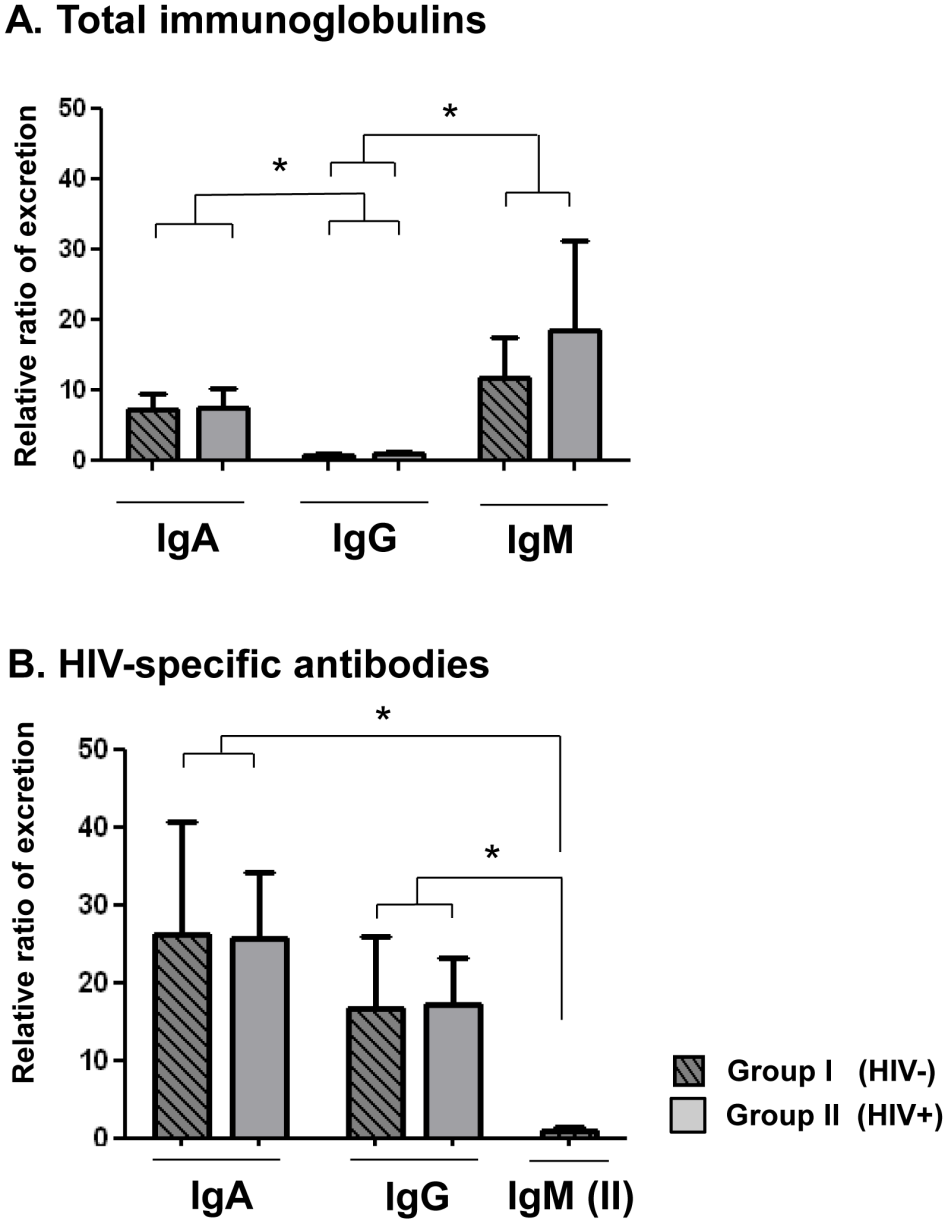
Relative ratios of excretion (RRE) by reference to lactoferrin of total IgA, IgG and IgM (A) and anti-gp160 antibodies of the IgA, IgG and IgM classes (B) in stool samples from8 HIV-1-infected mothers and their breast milk exposed non HIV-infected babies (group I) (grey bars) and from 6 couples of HIV-1-infected mothers and their breastfed HIV-1-infected babies (group II) (hatched grey bars). RRE is expressed as mean±standard error. The stars indicate the significant differences between the mean RRE of IgA, IgG and IgM, and those of HIV-specific IgA, IgG and IgM in the whole 14 study babies breastfed by their HIV-infected mothers (groups I and II) (* P<0.01).

The calculations of RRE by reference to Lf of stool immunoglobulins and HIV-specific antibodies in breastfed children from groups I and II are depicted in the Figure 2.

For stool immunoglobulins, the RREIgA,S (group I: 7.2±2.3; group II: 7.5±2.6; P>0.05) and RREIgM,S (group I: 11.7±5.7; group II: 18.4±12.6; P>0.05) were largely above 1, indicating intestinal synthesis of IgA and IgM. In contrast, the mean RREIgG,S (group I: 0.6±0.2; group II: 0.9±0.2; P>0.05) were inferior to 1, indicating that the intestinal production of IgG is likely less than the breast milk origin for this class. Similar observations were found for stools total Ig from HIV-negative control babies (data not shown).

For stool HIV-specific antibodies, the RRE were calculated when HIV-specific antibodies of the same isotype was detected in corresponding breast milk sample from the HIV-infected mother (denominator different of zero).Thus, the RREIgA to gp160,S (group I: 26.0±14.7; group II: 25.6±8.0; P>0.05), RREIgG to gp160,S (group I: 16.7±9.2; group II: 17.2±5.9; P>0.05) and RREIgM to gp160,S (group I: not applicable; group II: 2.7±0.2) were largely above 1, indicating intestinal synthesis of HIV-specific IgA, IgG and sometimes IgM. The local synthesis of HIV-specific IgA and IgG were 13- and 8- fold, respectively, higher than that of HIV-specific IgM (P<0.01). The levels of intestinal production of HIV-specific antibodies were similar in groups I and II whatever the class of immunoglobulins (P>0.05 for all comparisons). Taken together, the RRE calculations suggest that all babies exposed to HIV via breast feeding, infected or not, synthesize intestinal HIV-specific antibodies, mainly of the IgA and IgG isotypes.

### Functional Activities Against HIV-1 of Immunoglobulins Purified from Pooled Stools Samples of Children Breastfed by HIV-1-infected Milk

Two pools of stools samples from children of group I and from group II were constituted, in order to be subjected to immunoaffinity purification of total immunoglobulins. Resulting purified pooled stools immunoglobulins contained F(ab’)2 to gp160 (data not shown), showed similar SA [F(ab’)2 to gp160 purified from pooled stools of group I, 2.6±0.6 AU/µg, and of group II, 3.2±1.0 AU/µg; P>0.05)], and were used for further functional experiments.

**Figure 3:**
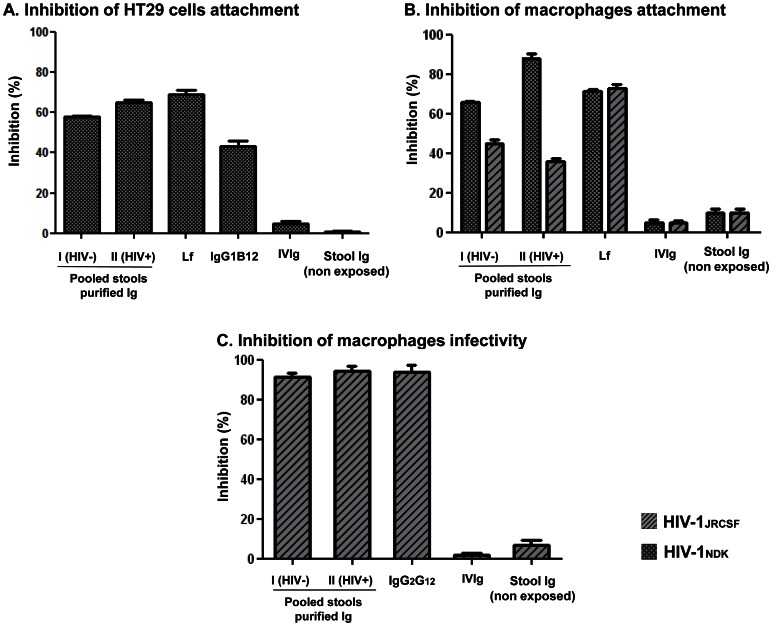
Functional activities against HIV-1 of immunoglobulins purified from pooled stool samples of children breastfed by HIV-1-infected milk. Immunoglobulins (Ig) purified by immunoaffinity from pooled stool samples from 8 HIV-1-infected mothers and their breast milk exposed non HIV-infected babies (group I) and 6 couples of HIV-1-infected mothers and their breastfed HIV-1-infected babies (group II), and containing F(ab’)2 to gp160 with similar specific activities, were constituted. **A and B.** Inhibition of the attachment of HIV-1NDKon HT29 cells and of HIV-1NDKand HIV-1JRCSF on monocyte-derived macrophages (MDM) by Ig purified from pooled stools of children breastfed by HIV-1-infected milk.HT29 intestinal cell lines and MDM were incubated with HIV-1NDKor HIV-1JRCSFin the presence of 30 (A) or 50 (B) µg/ml of pooled stools purified Igfor 1 hour at 37°C. Cells were then washed, lysed, and quantities of attached virus were evaluated by HIV p24 antigen measurement in the culture lysate using capture ELISA. Lactoferrin and the monoclonal antibody IgG1B12 were used as positive controls for inhibition; IVIg and total Ig purified from pooled stools of HIV non exposed, HIV-seronegative babies were used as negative controls. The experiments were carried out in triplicate with cells from three different donors. The HIV-1NDK or HIV-1JRCSF attachment inhibitions are expressed as percentage ± standard error of three independent experiments; **C.** Inhibition of the HIV-1JRCSF infection of MDM by Ig (50 µg/ml) purified from pooled stools of children breastfed by HIV-1-infected milk.The monoclonal antibody IgG2G12 was used as positive control for inhibition; IVIg and total Ig purified from pooled stools of HIV non exposed, HIV-seronegative babies were used as negative controls. The levels of viral production at day 6 postinfection were assessed by HIV p24 antigen measurement in the culture supernatants using capture ELISA. The experiments were carried out in triplicate with cells from three different donors. The HIV-1JRCSF infection inhibition is expressed as percentage ± standard error of three independent experiments. *Nota bene*. HT-29 epithelial cells were stained with mouse phycoerythrin (PE)-conjugated monoclonal antibodies anti-CD4 (Leu3a) (Becton Dickinson Biosciences, Mountain View, CA) and CXCR4 (12G5) (BD PharMingen, Le Pont de Claix, France), and with fluorescein isothiocyanate (FITC)-conjugated anti-human monoclonal antibodies DC-SIGN (DCN46) and CCR5 (2D7) from BD Biosciences (San Diego, CA) and GalCer (MAB342) (Chemicon International, Paris, France). Analysis was assessed using a FACSCalibur flow cytometer and CellQuest software (BD Biosciences). Results are presented as the percentage of receptor-positive cells. Forty-six percent of HT-29 cells expressed GalCer, 29% CXCR4 and 10% CCR5, whereas very low (<0.1%) expression of CD4 and DC-SIGN was detected.

The capability of purified pooled stools immunoglobulins to inhibit the attachment of HIV-1 on HT29 cells and on MDM was first evaluated. As shown in Figure 3A, pooled stools immunoglobulins(30 µg/ml) purified from group I and group II inhibited the attachment of HIV-1NDKon HT29 cells by 58.0±0.6% and 65.0±1.1%, respectively. Lf (50 µg) and the monoclonal antibody IgG1B12 used as positive control inhibited the attachment of HIV-1NDKon HT29 cells by 69.0±2.3%, and 43.5±4.5%, respectively. As shown in Figure 3B, pooled stools immunoglobulins(50 µg/ml) purified from group I and group II inhibited the attachment of HIV-1NDKon MDM by 65.7±0.9% and 88.1±2.3%, respectively, and the attachment of HIV-1JRCSF on MDM by 45.0±1.7% and 35.7±1.7%, respectively. Lf (50 µg) used as positive control inhibited the attachment of HIV-1NDKon MDM by 71.3±0.9%, and the attachment of HIV-1JRCSFon MDM by 72.7±2.0%. IVIg and total immunoglobulins purified from pooled stools of HIV non exposed babies, used as negative controls, inhibited the attachment of HIV-1NDKon MDM by 5.0±1.1% and 10.0±2.0%, respectively, and the attachment of HIV-1JRCSFon MDM by 4.6±1.2% and 9.7±2.3%, respectively.

The capability of pooled stools purified immunoglobulins to inhibit the infection of MDM by HIV-1JRCSF was further evaluated. As shown in Figure 3C, pooled stools immunoglobulins(50 µg/ml) purified from group I and group II inhibited the infection of MDM by 91.1±2.3% and 94.5±2.0%, respectively. The monoclonal antibody IgG2G12 used as positive control inhibited the infection of MDM by HIV-1JRCSF by 94.0±3.5%. IVIg and total immunoglobulins purified from pooled stools of HIV non exposed babies, used as negative controls, inhibited the infection of MDM by HIV-1JRCSF by 1.7±1.2% and 7.0±2.5%, respectively.

## Discussion

Identifying factors involved in decreasing HIV transmission via breast-feeding would provide important insights into the type of immune responses required to protect against infant HIV acquisition. In the present study, the B cell immune intestinal response to HIV was investigated using stool samples from breastfed infants born from HIV-1-infected women. The stools from non-infected as well as HIV-infected children exposed to HIV-1 via breast-feeding contained HIV-specific antibodies, mainly of the IgG isotype and to a lesser extent of the IgA isotype. The RRE calculations by reference to Lf suggested that all babies exposed to HIV via breast-feeding, infected or not, synthesized actively intestinal HIV-specific antibodies, mainly of the IgA and IgG isotypes. Furthermore, purified pooled stools immunoglobulins containing anti-gp160 antibodies demonstrated in vitro functional properties against HIV, by blocking the attachment of HIV-1 on epithelial (HT29) and monocyte-derived cells, and by inhibiting the viral infection of MDM. These findings demonstrate that an intestinal humoral immune response to HIV actively develops in infants born from HIV-1-infected mother and breastfed with HIV-infected milk, likely in addition with breast milk-derived passive seepage of ingested HIV-specific antibodies from the HIV-infected mother. The intestinal production of HIV-specific antibodies in HIV-exposed children via breast-feeding indicates that inductive sites of the afferent branch of the MALT in contact with ingested HIV particles coming from breast milk are immunized against HIV antigens, and that the intestinal sites of the efferent branch of the MALT actively produce HIV-specific antibodies released within the intestinal lumen. The in vitro blocking properties of HIV-specific antibodies purified from stools of children exposed to HIV via breast-feeding suggest that intestinal humoral immunity to HIV could be functional in vivo against the virus, resulting in hampering the possibility of infants’ infection.

The cofactors modulating HIV acquisition in the infant breastfed by HIV-infected mother have so far received until now relatively little attention [21], [23], [57], and few studies on this subject have been yet published [25], [26]. Our observations demonstrate for the first time the activation of HIV-specific humoral immunity by the intestinal mucosa of HIV-exposed children by breast-feeding. These findings are in keeping with previously published studies reporting activation of cellular or humoral immunity in breastfed HIV-exposed children. Thus, breast-feeding HIV-1-exposed uninfected babies frequently showed HIV-1-specific interferon (IFN)-γ responses [26]. Of more than 200 breast-feeding HIV-1-exposed uninfected babies who were serially and prospectively assessed for HLA-selected HIV-1 peptide-specific cytotoxic T lymphocyte (CTL) IFN-γ responses by means of enzyme-linked immunospot (ELISpot) assays, almost half had HIV-1–specific CTL IFN-γ responses despite the absence of HIV-1 infection [26]. These findings suggest that, rather than completely escaping viral exposure, many HIV-1–uninfected babies born to HIV-1–infected mothers are exposed to cell-associated HIV-1 and elicit immune recognition of HIV-1–infected cells. In addition, infant immune responses to HIV in saliva have been hypothesized [23]. Because secretory IgA is not transported actively across the placenta, levels are generally low to absent at birth and increase with age, achieving adult levels near 6–8 years [58]. In a prospective cohort study, Farquhar et al. explored whether HIV-1-exposed, uninfected babies make immune responses in saliva after natural challenge with maternal breast milk or cervicovaginal secretions containing HIV-1 [25]. Overall, only 8% of HIV-1-uninfected babies in the study tested positive for HIV-1-specific salivary IgA at one time-point, and all babies with IgA responses remained uninfected during 1 year of follow-up. While the proportion of babies with positive assays was relatively low, these results also support that salivary HIV-1-specific IgA can be elicited in babies by immunizing neonates, and provide some evidence that natural HIV-1 exposure via the oral route can stimulate a humoral immune response in babies younger than 6 months of age [25]. Taken together, our present observations on HIV-specific intestinal immunity, as well as those previously reported on HIV-specific CD8 cytotoxic immune responses and on specific antibody salivary production against HIV, strongly indicate that both acquired cellular and humoral immunity against HIV antigens occur in children exposed to HIV via breast-feeding. Although poorly studied until now, innate immunity in breastfed children born from HIV-infected mothers may also play an important role synergistic of adaptive immunity in preventing and containing HIV infection and protecting against immune-escape viruses generated by more narrow adaptive immune responses [57], [59], [60].

In a first approach, we analyzed quantitatively the nonspecific and HIV-specific humoral immune responses in the breast milk of infected mothers and in the stools of children exposed to HIV-infected milk, themselves infected or not with HIV. The levels of total immunoglobulins of unknown specificities were increased in the milk of HIV-infected mothers, mainly within the IgG isotype, indicating the activation of mammary production of B cell-derived immunoglobulins. In the stools of HIV-exposed children via breast-feeding, the levels of total immunoglobulins, mainly of the IgA and IgG classes, were increased by comparison with non HIV-exposed breastfed children. The interpretation of these observations is not unique. Increased concentrations of stool immunoglobulins may be in part due indirectly to higher ingestion of breast milk immunoglobulins whose levels are increased. In addition, the increased levels of stools immunoglobulins in these children continuously exposed to HIV antigens could result from chronic stimulation of intestinal B cell-derived humoral immune system, resulting in enhanced staged intestinal production of immunoglobulins. Several hypotheses may be raised especially considering that B cell abnormalities are an important immunological feature of HIV infection. Indeed, it is well established that HIV antigenic pressure and virus-induced immune activation lead to polyclonal B cell activation and dysfunction including hypergammaglobulinemia, increased expression of activation markers, and loss of memory B cells as well as serological memory [61]–[63]. Intestinal B cell activation in breastfed HIV-exposed children may therefore be caused by direct and indirect effects of the virus. Thus, HIV is known to directly induce *per* itself B cell activation [61], [64]–[66]. In addition, breast milk is a complex fluid containing various soluble factors with diverse immunomodulatory properties on the mammary production of antibodies [67], [68], including Th2 cytokines and soluble CD14, believed to mediate B cell growth and differentiation [69]. The B cell immunomodulatory cofactors are variously disturbed in breast-feeding HIV-infected mothers showing frequent altered levels [20], [70], that could subsequently affect the B cell-derived immune response of the intestinal mucosa in breastfed children.Finally, the B cell compartment in uninfected HIV-exposed children might also be affected by the altered maternal immune system, including maternal immune activation *in utero* [71], [72]. Whatever the possible assumptions, our observations indicate that the mucosal humoral immunity is strongly stimulated in the couple HIV-infected mother/HIV-exposed breastfed children.

We further explored the HIV-specific humoral immunity, both in breast milk of HIV-infected mother, and in stools of their HIV-exposed infants. In the present series, breast milk samples from HIV-infected mother contained high levels of HIV-specific antibodies, including mainly antibodies of the IgG isotype followed by antibodies of the IgA class, and rarely of the IgM class. These results are fully consistent with numerous previously published descriptive studies on antibodies against HIV in breast milk of HIV-infected women [2], [33], [37], [73]–[80], and indirectly validate the reliability of the immunochemical procedures used in the present study. Indeed, we confirmed that antibodies of the IgG class constitute the predominant isotype of HIV-specific antibodies in breast milk detected in nearly all samples [24], [33], [75], whereas HIV-1-specific secretory IgA is the second class present in breast milk, detected in differing proportions of breast milk samples, varying from 23% of 15 days postpartum breast milk and 41% of 18 months postpartum milk in the study by Van De Perre et al. [2], to 59% of women in the study by Duprat et al. [76], and to all samples studied by Becquart et al. [33]. The scarcity of secretory IgM to gp160 in mature, non colostral breast milk has been previously emphasized by Becquart et al. [33]. Similarly to breast milk samples, HIV-specific antibodies could be detected in nearly all stools samples from children of non-transmitting and transmitting mothers, with a marked predominance of IgG antibodies, followed by IgA antibodies, and very rarely IgM antibodies. The very high specific activity of IgG antibodies to gp160 in stools samples, 12 and 7 times much higher than that of IgA to gp160, is remarkable for a mucosal product (stools) in which IgA immunoglobulins predominate over IgG. More generally, our observations may be considered as consistent with the current conception regarding the HIV *env*-specific humoral response in mucosal secretions as being primarily of the IgG isotype. Indeed, lower levels of HIV-1-specific IgA antibodies compared to IgG antibodies have been reported in breast milk and other mucosal sites such as the genital tract, saliva, tears and duodenal fluid [77], [78], [81]–[88]. This is apparently surprising given that locally produced secretory IgA is the predominant immunoglobulin isotype in most mucosal secretions [89]. In animal models, low or absent IgA responses have also been described in HIV-1-infected chimpanzee [90] and simian immunodeficiency virus (SIV)-infected macaques [91], [92]. Thus, in striking contrast to other mucosae-encountered microbial infections, HIV-1 and SIV do not induce vigorous specific IgA responses in any body fluids examined. The mechanism involved in this selective suppression of HIV-1-specific responses in the IgA isotype in mucosal secretions remains yet unresolved, and may be fundamental to understand how HIV is capable to be shed in corporeal fluid, *i.e.* to be produced by mucosal reservoirs despite the existence of mucosal immunity against the virus [21].

The origin of humoral mucosal immunity to HIV in stools from breastfed children born from HIV-infected mother remains unknown. Antibodies in stools may *a priori* come from ingested breast milk which contains HIV-specific antibodies, and from the infants themselves. Thus, a certain proportion of stool antibodies or F(ab’)2 moieties should correspond to undegraded or partially degraded HIV-specific antibodies coming from breast milk intake. In addition, HIV-specific antibodies in stools may be either transferred from infant’s plasma by transudation or locally produced by plasma cells belonging to the MALT [22], that migrate to the efferent branch of intestinal mucosa from other inductive mucosal sites, in particular, the oral- and gut- associated lymphoid tissues [22], [93]. In the present series, all infants were less than 1 year old, thus were seropositive for routine HIV serology, whatever their infectious status regarding HIV. Thus, plasma-derived IgG may have passively transudated into intestinal secretions, and account for a certain proportion of HIV-specific IgG in stools. Furthermore, in order to evaluate the possibility of intestinal production of HIV-specific antibodies, the relative fecal/intestinal production of immunoglobulins and HIV-specific antibodies in feces from breastfed children was evaluated by calculating their RRE by reference to Lf as breast milk intake factor. The working hypotheses were that immunoglobulins and Lf are glycoproteins similarly degraded within intestinal chyle, and that the intestinal production of Lf is negligible in the absence of gastro-intestinal inflammation or gastro-enteritis in breastfed children [49], as in the infants carefully selected for the study. Assuming that the diffusion of milk immunoglobulins is similar to that of Lf, the RRE calculation likely enables to evaluate the partition between passive intake of immunoglobulins and intestinal local production. The RRE by reference to Lf is quite similar to the relative coefficient of excretion (RCE) previously proposed to evaluate the mucosal excretion of immunoglublins by reference to albumin [86], [94], [95]. For stool total immunoglobulins of unknown specificities, the RRE calculations indicated intestinal synthesis of total immunoglobulins, IgA and IgM, harboring RRE largely above 1, whereas the stools IgG appeared mainly passively deposited instead of locally produced. These latter observations are reminiscent of the previous demonstration using the RCE calculations by reference to albumin of active mucosal production of polymeric IgA and IgM within the jejunal secretions whereas IgG and monomeric IgA in the jejunal fluid are mainly plasma-derived, and thus of transudative origin [94]. Applied to HIV-specific antibodies, the calculations of RRE by reference to Lf for HIV-specific IgA, IgG and IgM were largely superior to 1, likely indicating that HIV-specific antibodies detected in stools are not only originating from a passive ingestion of breast milk antibodies, but rather from active intestinal secretions. Taken together, these observations indicate that the infant intestinal mucosa of HIV-exposed breastfed children, infected or not by HIV, likely secretes actively HIV-specific antibodies, and thus that the HIV-specific antibodies in their stools are locally-produced in addition with possible passive transudation from plasma for the IgG class. The inductive sites of HIV-specific antibodies intestinal production are unknown, but free HIV particles and cell-associated virus present in breast milk reach continuously the oral mucosa, the tonsillar tissue and the upper gastrointestinal mucosa of exposed breastfed babies, which are immunocompetent tissues including the Waldeyer lymphoid tissue and the Peyer’s patches belonging to the afferent branch of the MALT [22]. The effector sites of HIV-specific antibodies intestinal production remain similarly unknown, but may include the lymphoid tissue of the jejunal and colonic mucosae, which belong to the efferent branch of the MALT [22], [96]. Acquired intestinal immunity against viral antigens in children has been extensively described with other infections than HIV, for example after natural intestinal infections by rotavirus [97] or norovirus [98], or immunization with oral live attenuated or inactivated poliovirus vaccine [99], [100].

The functionality of purified pooled stools antibodies to HIV was finally investigated using *in vitro* assays. The inhibitory properties towards viral attachment to intestinal epithelial cells and MDM of antibodies purified from pooled stools were first assessed. In the present study, HT29 epithelial colon carcinoma cell line was used to mimic the initial contact of HIV with intestinal epithelial cells [101], as it is thought to occur during HIV exposition of breastfed infants. Because a high proportion of HT29 epithelial cells expressed CXCR4 [102], X4-tropic HIV-1NDK strain was used in attachment inhibition assay. In addition, MDM were chosen because intestinal macrophages are considered as prominent HIV-1 reservoir in chronically established HIV infection [103]. MDM were used in attachment and infectivity inhibition assays by X4-tropic HIV-1NDK or R5-tropic HIV-1JRCSF strains, since both viral phenotypes may be present in mucosal secretions [104]. Furthermore, R5-HIV-1-infected macrophages in breast milk may be the most likely cells to transmigrate across fetal oral and intestinal mucosal epithelia [105]. Pooled immunoglobulins purified from stools of infants exposed to HIV by breast-feeding, whatever the infant HIV status, inhibited the attachment of HIV-1NDK on HT29 cells by 63% and on MDM by 40%, and the attachment of HIV-1JRCSFon MDM by 77%. In addition, purified pooled stools immunoglobulins inhibited the HIV-1JRCSF infection of MDM by 93%. Otherwise, since the stools of children breastfed by their HIV-infected mothers likely contain a small proportion of HIV-specific antibodies coming from breast milk, it is also likely that stools HIV-specific antibodies may also harbor other functional inhibitory properties against HIV, such as those previously demonstrated for breast milk specific antibodies, like HIV-1 transcytosis inhibition [37], [106], virus neutralization and antibody-dependent cell cytotoxicity [79]. Taken together, these observations demonstrate that HIV-specific antibodies purified from stools of HIV-exposed breastfed children harbor in vitro major functional properties against HIV.

The correlates of protection against mucosal acquisition and control of HIV-1 infection have not yet been clearly defined. Humoral factors, innate immunity, and specific antibodies present in external secretions, as well as cytotoxic lymphocytes distributed in mucosal tissues, have been considered as involved in the prevention and the local limitation of HIV at mucosal sites of viral entry, especially in exposed non infected individuals [107], [108]. The active intestinal synthesis of HIV-specific antibodies in HIV-exposed, breastfed non infected infants is reminiscent of the genital and oral mucosal immune response against HIV-1 in exposed uninfected individuals [108]–[111]. The genital secretions collected from HIV-1-exposed but persistently seronegative female sex workers do not however contain uniformly and easily detectable HIV-1-specific antibodies or neutralizing activities, and the existence of adaptive HIV-specific humoral immunity in genital mucosae exposed to HIV via sexual intercourse remains controversial [112]. The possibility of intestinal production of HIV-specific antibodies in HIV-exposed, breastfed non infected infants may therefore constitute a particularly relevant model of natural protection against HIV acquisition via mucosal routes. Indeed, despite the infants’ daily exposure at their oral and gastrointestinal mucosae to high amounts of cell-free and cell-associated HIV, the virus acquisition in breastfed children occurs infrequently. The puzzling fact that the majority of breastfed infants born from HIV-infected mothers remain uninfected, even after several months of breast-feeding, constitutes one of the major paradoxes of HIV transmission via breast milk. The infrequent breast milk HIV transmission despite prolonged exposure suggests that anti-infective properties of breast milk may play a relevant protective role against infection of exposed children, and that natural and/or resistance to HIV in breastfed children could also be involved [21]. Finally, our observations raised acutely the issue of the putative roles of functionally active HIV-specific, intestinal antibodies in HIV transmission through breast-feeding. In the present series, the HIV-specific intestinal immunity appeared qualitatively, quantitatively and functionally similar in HIV-infected as in non HIV-infected breastfed children. These features do not exclude a protective role in vivo of the adaptive HIV-specific intestinal immunity. Indeed, intestinal immunity to HIV developing after exposition to HIV-infected milk should be envisioned in the context of the multiple factors involved during HIV transmission via breast-feeding, which is in essence a multifactorial process [21]. During the duration of exposure, HIV transmission by breast-feeding may be associated with an imbalance between virus ingestion and mucosal immune response directed against the virus, and finally between infective and anti-infective properties of breast milk [20], [57] and exposed infant’s resistance [25], [26]. For example, in case of contamination during acute mastitis in the mother, the potentially protective intestinal immunity directed to HIV would have been insufficient to protect against exposure to large quantities of virus occurring therefore transiently [113].

Finally, our observations constitutes the basis to further investigate possible immunological correlates of protection in HIV transmission via breast-feeding that could be noteworthy for the design of a prophylactic vaccine. In developing countries notably in sub-Saharan Africa, the postnatal mother to child transmission of HIV-1 may continue even in the setting of optimal antiretroviral therapy [15]. For this reason, the development of new immunological strategies to reduce residual postnatal transmission remains important in order to improve survival of babies born from HIV-infected mothers in the developing world. From the above results, we conclude that the intestinal mucosa of children exposed to HIV by breast-feeding actively secretes HIV-specific antibodies harboring in vitro functional properties against HIV, and thus possibly protective in vivo. In vaccine perspective, the demonstration of adaptive intestinal humoral immunity in infants orally exposed to viral antigens provides the rational basis for attempting to develop a mucosal prophylactic vaccination by intestinal immunization against HIV in children orally exposed to the virus.
